# Investigating responses to object-labels in the domestic dog (*Canis familiaris*)

**DOI:** 10.1038/s41598-023-30201-1

**Published:** 2023-02-23

**Authors:** Hanna Kőszegi, Claudia Fugazza, Lilla Magyari, Ivaylo Borislavov Iotchev, Ádám Miklósi, Attila Andics

**Affiliations:** 1 Department of Ethology, ELTE, Eötvös Loránd University, Pázmány Péter sétány 1/c, Budapest, 1117 Hungary; 2 Department of Animal Breeding, Nutrition and Laboratory Animal Science, University of Veterinary Medicine, István utca 2, Budapest, 1078 Hungary; 3 Department of Social Studies, Faculty of Social Sciences, University of Stavanger, Stavanger, Norway; 4 Centre for Reading Education and Research, Faculty of Arts and Education, University of Stavanger, Stavanger, Norway; 5 MTA-ELTE “Lendület” Companion Animal Research Group, Hungarian Academy of Sciences – Eötvös Loránd University, Pázmány Péter sétány 1/c, Budapest, 1117 Hungary; 6 MTA-ELTE “Lendület” Neuroethology of Communication Research Group, Hungarian Academy of Sciences – Eötvös Loránd University, Pázmány Péter sétány 1/c, Budapest, 1117 Hungary; 7 ELTE NAP Canine Brain Research Group, Pázmány Péter sétány 1/c, Budapest, 1117 Hungary; 8 ELTE NAP Comparative Ethology Research Group, Pázmány Péter sétány 1/c, Budapest, 1117 Hungary

**Keywords:** Social evolution, Evolution, Psychology

## Abstract

Since the dawn of comparative cognitive research, dogs were suspected to possess some capacity for responding to human spoken language. Neuroimaging studies have supported the existence of relevant mechanisms, but convincing behavioral performance is rare, with only few exceptional dogs worldwide demonstrating a lexicon of object-labels they respond to. In the present study we aimed to investigate if and how a capacity for processing verbal stimuli is expressed in dogs (N = 20), whose alleged knowledge of verbal labels is only backed-up by owner reports taken at face value, and concerning only a few words (on average 5). Dogs were tested in a two-choice paradigm with familiar objects. The experiment was divided into a cue-control condition (objects visible to the owner vs. shielded by a panel, thereby controlling the owner’s ability to emit cues to the dog) and a response type condition (fetching vs. looking). Above chance performance in fetching and looking at the named object emerged on the level of the sample as a whole. Only one individual performed reliably above chance, but the group-level effect did not depend on this data point. The presence of the panel also had no influence, which supports that performance was not driven by non-verbal cues from the owners. The group-level effect suggests that in typical dogs object-label learning is an instable process, either due to the animals primarily engaging in contextual learning or possibly analogous to the early stages of implicit, statistical learning of words in humans and opposed to the rapid mapping reported in exceptional dogs with larger passive vocabulary.

## Introduction

The notion that dogs may have a passive understanding of spoken human language is one of the oldest ideas in ethology. Tests on a dog alleged to understand words were conducted already in the late twenties^1^. Konrad Lorenz, one of the founding fathers of ethology, further propagated this notion in his book “So kam der Mensch auf den Hund”^2^. He alleged, not shy of controversy, that they may even understand “every word.”

Today we see solid evidence emerging for a more nuanced version of this statement. However, there is a crucial gap between the behavioral and neuro-physiological sources. While neuro-imaging studies suggest mechanisms relevant for the identification and processing of cues derived from spoken language among randomly selected samples of dogs^3–5^, only a few individuals worldwide have actually shown a convincing behavioral demonstration of label-object associations, further on referenced as exceptional or *Gifted Word Learner* dogs^6–11^.

Randomly selected dogs do not learn object labels, even after months of intensive training^9^. An earlier studied sample of dogs also failed to learn object labels during a 4-month period, but successfully acquired action commands^12^. Connecting labels to objects seems to be a particular challenge for the species, justifying a distinction between exceptional and typical dogs, where exceptional dogs appear to learn object labels effortlessly, while typical dogs do not show evidence of learning, even if exposed to extensive training. The asymmetry between how most dogs learn object versus action words may be traced to several, not mutually exclusive explanations, listed below.

- (1) *State-dependent learning*: What most dogs learn when interacting with objects may not generalize across contexts. State-dependent learning dominates word acquisition in humans up to the age of 4 years^13^.
- (2) *Spatial bias*: Dogs generally appear to have a bias about processing information as being about location rather than objects^14–17^. This spatial bias is expressed less in dogs with high scores on g (general cognitive ability,Iotchev et al. in prep.) thus it can be expected that differences in learning capacity and strategy could underlie the ability to bind labels and objects.
- (3) *Implicit versus explicit learning*: Gifted Word Learner dogs display a fast acquisition, ranging from as few as four exposures in socially motivating contexts^8^ to possibly even one-trial learning^7^. Language acquisition in humans begins, however, with statistical learning^18^, which is implicit, i.e. outside awareness, in its early stages^19^. Faster, specifically one-trial encoding, on the other hand, directly leads to the formation of episodic memories which support conscious recollection in humans^20^ and possibly animals^21,22^. Importantly, neural markers of statistical learning precede explicit knowledge of the learning material as measured through a discrimination choice task^23^. Therefore the explicit stage may not be required to see changes in the organism’s reaction to the learning target. This implicit knowledge, however, is likely less stable since one possible mechanism by which information in the brain is precluded from reaching awareness is a low signal-to-noise ratio^24^.
- (4) *Effects of prior knowledge*: Object-labels themselves increase the efficiency and strength of object representations^25^, thus we can assume that implicit learning needs to cross a threshold and a few object-label associations need to be acquired before rapid learning of a great number of words can take hold.

On the basis of these explanatory accounts, it can be expected that word learning in typical dogs may not be easily detected on the level of the individual. Instead we might observe a group level effect suggesting instable associations and a more implicit level of acquisition. Performance based on implicit and/or instable associations might furthermore be easily masked by attentional biases, like preferring to attend to one side (left versus right) or a particular object. Other factors that may render word representations instable stem from deficiencies in processing phonetic and contextual information relevant during acquisition^26,27^.

To understand how typical and exceptional dogs differ, focussing on either Gifted Word Learners or completely naïve dogs may limit our scope, since the former easily progress to acquire hundreds of names, while the latter have so far not been trained successfully to learn object labels in an experimental setting^9,12^. Therefore, in the present study, we selected dogs who, according to their owners, respond to at least two object names. The sample’s average performance, however, did not exceed 5 labels, while dogs suspected to be in fact Gifted Word Learners (see below), were excluded in a series of post-hoc control analyses. We aimed to test to what extent these dogs could differentiate between any two objects by their name alone, i.e. excluding the possibility that accompanying gestures guided their responses in a two-way choice task. We therefore controlled what owners could see and thus signal unintentionally by introducing two cue-control conditions, defined by the presence/absence of a panel which blocked the owner’s view of the objects. Studies furthermore show that dogs’ knowledge in some tasks may guide their gazing direction, but not their fetching responses [see e.g. Kubinyi et al.^28^ and Gábor et al.^29^]. This is likely because physical responses like approaching or fetching can be guided by factors other than stimulus identity, e.g. the animal’s preference for a given stimulus independent of the task-goal. Therefore, two response type conditions were paired with each cue-control condition: fetching task and a passive condition that allowed to measure dogs’ gazing orientation and duration (later on referred as ‘looking’ condition). If dogs are able to choose objects only by hearing the corresponding labels, they should perform above chance in both conditions. If dogs rely on non-verbal cues for choosing between objects, their performance would be affected by the presence of the panel and they could perform above chance only in its absence. Dogs were expected to fail (respond at chance level) in both cue-control conditions, if neither labels nor gestural cues were acquired.

## Methods

### Ethical statement

Our experiment is based on non-invasive procedures for assessing dogs’ behaviour. According to the ethical statement issued by the Hungarian “Scientific and Ethical Committee of Animal Experiments” (PEI/001/1056-4/2015) and the corresponding legal definition, in Hungary, this non-invasive study is not considered an animal experiment. All owners gave written consent to participate with their dogs in the study.

### Subjects

Twenty adult dogs (age: 2–11 years, M ± SD = 5.1 ± 2.5 years, 9 females; detailed list in Supplementary Table S1) of various breeds (11 different breeds and 2 mixed-breed dogs) participated in the study. According to their owners they were able to fetch at least two objects reliably by their names. The maximum number of known objects reported for a dog in this sample was 16, M ± SD = 4.8 ± 3.4 objects. Since the inclusion of Gifted Word Learners was difficult to prevent with this requirement, the best performing animal was excluded from some of our analyses (elaborated below). Dogs meeting above criterion were collected through online questionnaires, advertisements and personal contacts. None of the dogs had received extremely regular or intensive training regarding object labels prior testing. All dogs participated in all parts of the experiment.

### Stimuli

For each dog two objects were selected. These were taken from the owner’s household, among the objects the dog was claimed to identify by their label. They were used during all parts of the experiment. The owners were suggested to choose two objects (if there were more options) which were possibly equally preferred by the dog, and similar in size (fetchable). The objects differed across dogs and included balls, ropes, frisbees, socks, slippers, plush toys, etc. however, the verbal label used by the owners could be the same (e.g. “labda” for a ball, in the case of 9 dogs).

### Experimental setup

The experiment was carried out in the laboratory rooms at the Department of Ethology, ELTE, Budapest. All sessions were recorded with digital cameras.

**Figure 1:**
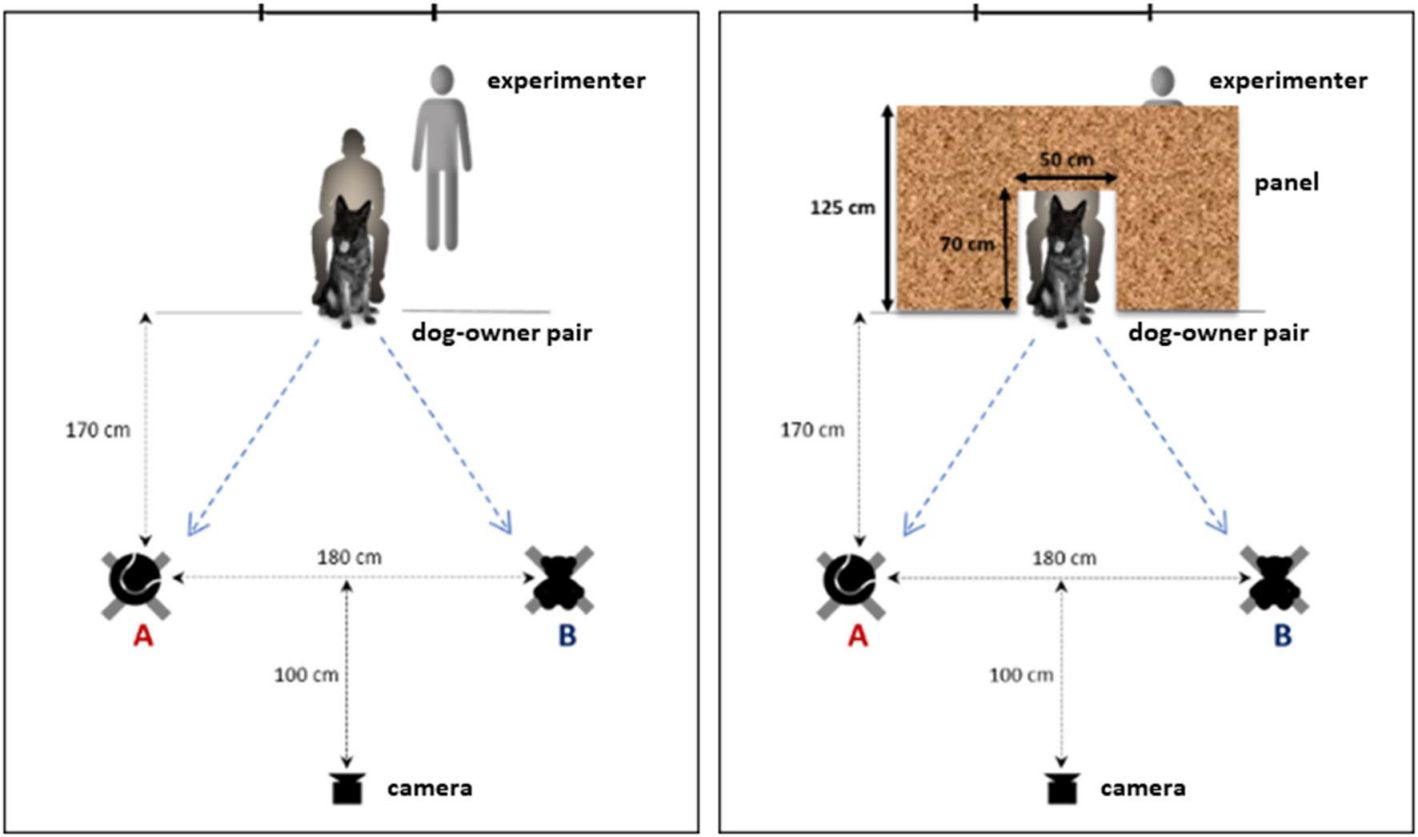
Experimental setup displaying the two cue-control conditions. Left image shows the setup without panel, right image shows the setup with panel between the owner and the objects. Object positions are indicated with A and B.

The two objects were positioned on the floor opposite the dog, in 170 cm distance, 180 cm from each other, on the left and right hand-side. The camera was placed on a tripod facing the dog, in 100 cm distance from the line of objects. In one condition a panel (height: 125 cm, opening: 70 × 50 cm) was also present (Fig. 1). Position of the objects (left/right) was set by the experimenter according to a predefined semi randomized schedule before every two trials, positioning one object after the other, while the dog was sitting by the owner.

One dog was tested at his home, with the only difference that the panel was substituted by a scarf on the owner’s eyes.

The dogs were tested in two cue-control conditions. In one condition there was a panel between the owner and the objects (panel condition), while there was no panel in the other condition (no panel condition). In the no panel condition both the dog and the owner could see the objects, while in the panel condition the presence of the panel prevented the owner from seeing the objects, therefore the choice behavior of the dogs could not have been influenced by the owner using non-verbal cues.

Additionally, the dogs’ reaction to the spoken words was observed in two response type conditions: a fetching and a looking condition. During the trials of the fetching task the dog was released after each request and allowed to fetch an object, while in the looking trials it was held by the owner, and only the gaze/head turn toward the objects and looking duration were measured.

Deriving from the combination of the two response type conditions and the two cue-control conditions the test for each dog consisted of four sessions (fetching – no-panel, fetching – panel, looking – no panel, looking – panel). The sessions were carried out on two different occasions (two per occasion, each on a separate day) to maintain the dog’s motivation level. The different sessions followed each other in a randomised order across dogs (see Supplementary Table S2) to control agains an order effect (e.g. performance in the fetching condition being affected by experience in the looking condition). Sessions defined by the same cue-control level (panel or no panel) always followed each other on the same occasion. Each testing occasion lasted about 30 min, including a 10-min-break between the two sessions. The structure of a session is outlined below (in *Procedure*).

### Procedure

Within each session each object was requested in three test trials out of six in total, resulting in a semi-random trial sequence concerning target object identity. The same object was asked maximum twice in a row. During each trial the experimenter stood slightly backwards next to the owner, and left this position only after the trial ended.

At the beginning of each session the dog and owner entered the room together with the experimenter. The dog was released to explore the room while the experimenter gave instructions to the owner. We asked owners to sit on a chair throughout the experiment, hold the dog at the beginning of each trial, then request the one object in each trial we instructed them to request, by either saying out loud the name of the object or embed the name in a sentence such as “Where is the < object name > ?” or “Fetch < object name > !”. The exact verbal command depended on how the object was usually requested at home. In the fetching response condition, we asked them to release the dog after the request. In the looking condition, we asked them not to release the dog after the request but hold them and praise them a few seconds after the request. In all conditions owners were instructed to avoid gesturing or providing other visual cues to their dogs.

Four motivational trials were implemented before every two test trials, where only one object at a time was available and the dog was rewarded with food for fetching it. Both objects were placed at both of the positions one after the other and dogs were requested to fetch it in the same way as during the fetching trials. With only one object being on the floor at a time, there was no chance of error in these trials. This served the purpose of maintaining motivation, by enabling the dog to physically interact with the objects and by rewarding its responses.

After the motivational trials, before the two test trials, when both of the objects had already been positioned, the experimenter took the dog on leash and showed it each of the objects one after the other, to ensure that the dog had seen the position of each object before the trials started. Since the order in which objects were shown was fixed, but the identity of the target objects switched regularly (see above), the position of the target object and the position of the last shown object overlapped on average 50% of the time.

Each test trial started with the dog sitting in front of or in some cases, next to their sitting owner, with the two objects placed on the floor. In each trial, according to a pre-made, semi-randomised order, one of the objects was requested by the owner.

In the fetching response condition, the dog was released after the request at the beginning of the trial and owners were asked to praise the dog after fetching any object. The chosen object was then placed back on its initial position by the experimenter for the second trial.

In the looking condition, the dog was held by the owner during the whole trial. The owner praised the dog a few seconds after the request, whatever direction it looked during that time. The subsequent trial followed without the dog being released to manipulate the objects inbetween. Initial looking direction was not controlled for, thus it was possible that the dog was already looking at one of the objects before the request was made. One dog showed signs of frustration when she could not fetch the objects, therefore, during the second looking session she was released a few seconds after the object label was uttered (the first look before the release was used for analysis, therefore this did not affect her results).

### Behavioural variables and statistical analysis

Only test trials were analysed. Behavioural data was collected from subsequent video analysis in Solomon Coder SOFTWARE (Version Beta 17.03.22). The coded variables are described below (see Table 1). Interrater reliability was tested for the data of 5 dogs and the variables ’Look before’ (Kappa coefficient (unweighted): 0.7709), ’First look’ (Kappa coefficient (unweighted): 0.7523), ’Duration “A”’ (Pearson’s correlation: r = 0.8840803, t(58) = 14.407, p-value < 0.0001), ’Duration “B” (Pearson’s correlation: r = 0.9446263, t(58) = 21.923, p-value < 0.0001), ‘Object Fetch’ (Kappa coefficient (unweighted):1).

**Table 1:** Coded variables.

Coded variables	Definition	Value	Type of trial
Look before	Toward which object the dog was looking one second before the request	A/B/0	*Fetching* + *Looking*
First look	Toward which object the dog was looking first after the request (for at least 0.2 s)	A/B/0	*Looking*
Object fetch	Which object the dog took back to the owner	A/B/0	*Fetching*
Duration looking	How long the dog looked at object A versus B during 2 s after the owner’s request, counting from the first orienting to one of the objects	s	*Looking*
Side	Which side the dog went first/looked for most of the time during the 2 s	R/L	*Fetching* + *Looking*

The two choice objects are indicated below by A or B. If no object was fetched/attended a zero was scored for that trial. Directions, right and left, are indicated with R/L, respectively.

“Object Fetch” was analysed only in the fetching task, “First Look”, “Duration A” and “Duration B” were analyzed only in the looking task, while “Look Before” and “Side” was analyzed in both tasks. The analysis of the fetching performance was focused on the “Object Fetch”, while analysis of the looking performance was focused on the “First Look” variable.

First Look (binary variable) was coded as correct when the time spent looking at the requested object was at least 0.2 s. If the dog looked back at the owner when the object label was uttered and thus might have looked at an object for shorter than 0.2 s because of turning its head, the trial did not count for measuring “First Look”. The instruction for the owner was that she should request the object only once in a trial. However in a few cases, owners repeated the requests multiple times. In these cases the coding of the 2 s period started when the dog first looked toward the objects, and subsequent requests were not taken into account.

Object Fetch (binary variable) was coded as correct when the object taken and brought to the owner was the object named in the request.

Success (derived from Object Fetch and First Look, binary variable): In case of the fetching task, a test trial was counted as successful (coded by 1) if the dog fetched the requested object, and counted as unsuccessful (coded by 0) if the dog fetched the wrong object. Trials were considered invalid if the dogs made no choice after several requests, but had a visible reaction, i.e. startled, moved, oriented after hearing the request (4 out of 240 trials, 1.67%). Completely omitted from analysis were trials in which dogs did not respond in any way to the commands, since these may tap on the animals’ attention rather than their capacity for verbal labels. In case of the looking task, a test trial was counted as successful (coded by 1) if the dog looked first at the correct object and counted as unsuccessful (coded by 0) if the dog looked first at the wrong object. Trials when the dog had no reaction at all or looked at the objects for less than 0.2 s were excluded from the analysis (13 out of 240 trials). Of all dogs 100% had only valid trials in the fetch + panel condition pair, 95% in the fetch + no panel condition pair, 80% in the look + panel condition pair, and 70% in the look + no panel condition pair.

The Object Bias Index and Side Bias Index (derived, numeric) were calculated for each occasion, independent of task (looking versus fetching), but across trials defined by the same cue-control level (panel versus no panel). The difference between the occurrence of the majority response and the less frequent response was divided by the total number of responses (for Object Bias: preferred object—non-preferred object/total; for Side-Bias preferred side—non-preferred side/total).

Statistical analysis was implemented in RStudio (Version 1.1.453). We applied generalized linear mixed-effect models (GLMMs) using the lme4 package. In all models, ID (dog’s name) was included as a random grouping factor. First, we tested whether the cue-control condition (panel vs. no panel), the response condition (fetching vs. looking), and their interaction influenced Success (calculated on the basis of First Look and Object Fetch). The model was optimized by removing non-significant factors (starting with the interaction) and an intercept-only model was used to test for above chance performance. The latter was to be applied separately to the looking and fetching performances and/or to the panel versus no panel conditions if the effects of the fixed factors or their interaction would be significant, and over the entire data if not.To evaluate the effect of object and side bias on the performance, Object Bias Index and Side Bias Index were subsequently included as explanatory variables in a GLMM model. Initial looking direction was evaluated by analysing the “Look Before” variable, using paired *t* test to compare the frequencies of looking at the later named and unnamed object before the request. Additionally, the durations of looking at correct and wrong objects were analysed by paired *t* test, using the average times spent looking on the correct and wrong objects during the looking trials in case of each dog. Dogs’ performance, as well as object and side bias were evaluated on the individual level as well.

Additional post-hoc analyses (one sample *t* tests and correlations) were performed in SPSS v25. The one-sample *t* tests were performed in order to control for the possibility that pooling together data across conditions may not be sufficiently justified by the statistical arguments (i.e. by the absence of an interaction in the GLMM). Note, for example, that trials with and without the panel come from different occasions/days. Whereas Success was included as a binary variable in the GLMMs, in the t-tests we used ratios (correct/valid trials).

## Results

**Figure 2:**
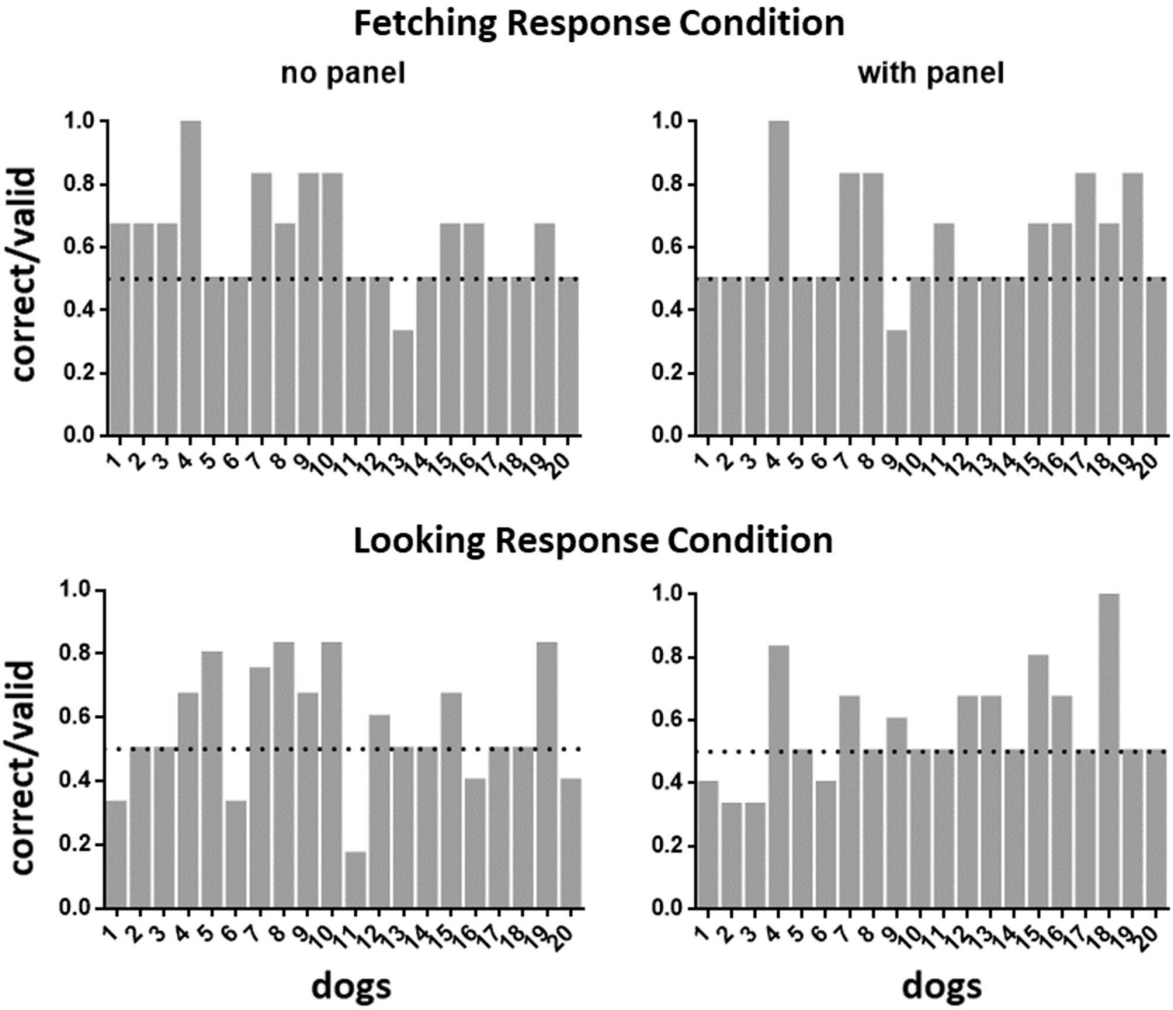
Overview of the proportion of correct choice trials (divided by total number of valid trials, dotted line indicates chance level) for each of the 20 dogs in both response conditions, with and without the panel.

A GLMM including the factors cue-control (panel versus no panel), response condition (looking versus fetching) and their interaction, with Success as the target variable, was run first to test for a possible interaction. Since there was no significant interaction effect (p > 0.9) we proceeded to test the fixed effects in a separate model. Neither cue-control nor response type condition had a significant effect (p > 0.3). Finally, Success was tested against chance-level (0.5) by using an intercept only model. This effect was significant (binomial GLMM, estimate (**β**_**0**_) = 0.374, z-value = 3.737, p < 0.001). Performance in the fetching and looking response conditions is summarized in Fig. 2.

Although the null effect of interaction in the GLMM suggests the same group-level effect across cue-control conditions, we performed post-hoc one-sample *t* tests to verify if for each response type + cue-control condition-pair choice performance was significantly above chance. The effects were significant for the fetching + no panel condition pair (t_19_ = 3.470, p = 0.0026) and the fetching + panel condition pair (t_19_ = 3.036, p = 0.0068). No significant difference was found for the looking + no panel and looking + panel condition pairs (p > 0.05).

We then also tested if the owner estimates for the dogs’ object-label knowledge bears any relationship with their performance by correlating the number of correct choices from each condition pair with the number of object-labels that were claimed the dogs could understand. There was a significant correlation between claimed capacity and correct choices for the condition pair fetching response + panel (r = 0.478, p = 0.033). No other correlation was significant (p > 0.2).

Next, dogs were tested individually for bias in their responses. Six dogs showed significant side preference in the fetching response condition, and two dogs in the looking condition, while 11 dogs showed significant object preference in the fetching response condition and 6 dogs in the looking condition (binomial test, p < 0.05). We conducted a GLMM containing “Success” as the dependent variable, and the previously calculated “Side Bias Index” and “Object Bias Index” as explanatory variables to test the effect of these biases on the animals’ performance. A significant negative effect was found for the Object Bias Index (estimate(**β**_**1**_) = − 0.9843, z-value = − 3.297, p < 0.0001) and the Side Bias Index (estimate(**β**_**2**_) = − 0.7750, z-value = − 2.430, p = 0.0151). The intercept also was significant (**(β**_**0**_) = 1.2354, z-value = 4.915, p < 0.0001).

Initial looking direction was not controlled for in above analyses. An additional analysis of the “Look Before” variable, however showed that there was no significant difference in the frequency of looking at the named object versus the unnamed object, before the request was made (paired *t* test, t_18_ = 0.7441, p = 0.4664).

An additional group level analysis of looking durations in the looking condition revealed that dogs spent significantly more time looking at the correct objects (M = 0.62 s) than at the wrong objects (M = 0.46 s, paired t-test, p = 0.027).

Individual performance based on the ‘Object Fetch” and “First Look” variable respectively showed that only one dog performed significantly above chance in the fetching response condition (binomial test, p = 0.039), while none of the dogs performed above chance in the looking condition.

Excluding this dog, overall group level performance remained significant (GLMM, estimate (**β**_**0**_) = 0.3341, z-value = 3.4600, p = 0.0005). The effects of the post-hoc tests for each response + cue-control condition-pair were significant for the fetching + no panel condition-pair (t_18_ = 3.153, p = 0.0055) and the fetching + panel condition pair (t_18_ = 2.721, p = 0.0140). Post-hoc test effects were not significant for the looking + no panel and looking + panel condition pairs (p > 0.1).

## Discussion

In the present study, selecting non-exceptional dogs with basic object label familiarity (dogs who respond to at least two object labels according to their owners) revealed a low incidence of stable word knowledge among typical dogs. Only one of the dogs was choosing above chance in our two-way choice tests. This is in sharp contrast with the few outstanding Gifted Word Learner dogs reported in the literature so far, which can learn the names of hundreds of toys^7–9,11^ and reliably respond to these labels above chance.

The dogs in the present study performed two types of object-choice (fetching and looking) following verbal instructions. Although individual performances were below threshold, the animals performed above chance across both response conditions when analysed as a group. It can be excluded that the group-level results were driven by the single above-chance performance of one dog, since there was no change in significance level for the fetching response condition when this individual was removed from the sample and for the looking response condition a significant effect changed to a trend. A possible responding to gestural cues can also be excluded since the presence of the panel had no effect in our analyses.

Our post-hoc tests for each response + cue control condition-pair separately lend additional support for the panel playing no role in fetching choice performance, as it remained significant for each cue-control condition. However, the post-hoc analyses do suggest that fetching performance was the more reliable read-out variable in this study compared to looking performance, which was not significant when analysed separately for each cue-control condition. Although other studies found looking behavior to be more sensitive for the tested capacity, e.g. dogs’ responding to expressed human preferences^28^, object-label associations might be more ‘embodied’ in active behavior, which is also in line with the finding that dogs learn responding to action words more readily^12^. It is also likely that many dogs acquire these associations during fetching games with their owners.

We also performed a set of correlations for each condition-pair (response and cue-control conditions) to inquire the reliability of owner estimates for predicting the actual behavior of the dogs. Only for the fetching + panel combination was the estimate given by the owners predictive of the number of correct choices, suggesting some external validity for the owner-given reports. That the correlation only reached significance with fetching is not surprising given that fetching was generally linked to more correct choices. The role of the panel is less straightforward. It could suggest that owner’s uncontrolled reactions to the sight of the objects is not uniformally helpful to the animals, but can also be a misleading cue.

We had specified four factors which may weaken the consistency in animals’ responding, even when, as the group-level effects suggest, associations are present. These were, in short: state-dependent learning, spatial bias, implicit learning, and prior knowledge. We observed some indication for the role of prior knowledge, i.e. the effect which already acquired labels appear to have on object-related cognition and learning^25,30^. Specifically, the owner-reported “vocabulary” of the dogs correlated with their performance, although in only one of four cue-response conditions. A direct demonstration of statistical learning, the role of context, or the possible effect of cognitive biases that could slow down object-centred associations (see e.g. Iotchev et al. in prep.) cannot be demonstrated with the current design. Instead, the present results suggest that biases for side and objects might have masked the individual performances in the sample and leave open the possibility that the lack of individual success is a matter of performance-impairing bias rather than missing knowledge. Future work will need to directly address factors that could impact performance in such tasks on a more cognitive level, such as the stage of learning (implicit versus fast mapping) or cognitive biases [e.g. spatial bias, as observed in^14–17^]. This will shed more light on how capacities relevant to processing verbal stimuli^3–5^ eventually unfold into a reliable use of labels for objects, as seen in Gifted Word Learner dogs^6,8^. The effects observed here for side and object bias are also not mutually exclusive with the hypothesis of instable associations. Inability to compete with distractors would be expected for implicitly stored information, since it is likely implicit by means of a lower signal-to-noise ratio [discussed e.g. by Phillips^24^ in the context of unconscious “perception”].

Individually, one dog (Demi) performed above chance during the fetching response condition, demonstrating a completely balanced, stable performance, fetching the correct object 5 out of 6 times in both conditions. Interestingly, in her case the number of objects reportedly known were also much higher (16 objects), than for the remaining dogs. One dog (Borisz) performed without mistake in the first fetching session (with panel), but he refused to fetch any object in the last 4 trials of the second session (without panel). This further supports the earlier suggested possibility that reactions from the owner when they can see the objects may at times be misleading or confusing to the animal. We also cannot exclude lowered motivation playing a part, although we had attempted to counter this by spreading the experiment over separate occasions. For a number of dogs (11 in the fetching condition, 6 in the looking condition) one object was consistently preferred over the other. These preferences may have emerged during the testing sessions or, alternatively, they could have been already present, without the owners detecting those. Future studies may want to determine object preference with pilot tests, as in e.g. Kubinyi et al.^28^. Zee, Zulch and Mills^31^ report in a case study of a single, word-knowledgeable dog, that a third kind of bias may affect choice behavior in testing object-label associations. It concerns object features like size and shape (only the former was directly demonstrated in their study), rather than the animal’s individual preference for e.g. playful interactions. Similarity in size, however, was one of our criteria for choosing the objects to be used during testing.

Individual performances in this study were mostly below chance, indicating that the formation of object-label connections is not solid in most of the tested animals. It is also worth noticing that a more systematic and/or intensive training has not been beneficial in previous attempts of teaching object labels to dogs^9,12^, thus it is possible that this proposed implicit stage of learning is an upper limit in the case of most typical dogs. This upper limit may also reflect a state-dependent learning approach to object-label acquisition^32–34^.

The possibility that object-label learning is in an implicit acquisition stage in most dogs should be tested more directly in the future. This may be aided by monitoring changes in neural markers of learning, as common in the literature on neural entrainment in humans^35,36^ and relate them to neural and behavioral correlates of awareness, e.g. stable performances and widespread cortical synchronization.

## Supplementary Information

Supplementary Information 1.

Supplementary Information 2.

Supplementary Video 1.

Supplementary Video 2.

## Data Availability

The data used here is attached as a supplementary file, additional information will be shared upon request by the corresponding author: Ivaylo Borislavov Iotchev, PhD, ivaylo.iotchev@gmail.com.
