# Supplementary material for: Investigating responses to object-labels in the domestic dog (Canis familiaris)
Source: Sci Rep. 2023 Feb 23;13:3150. doi: 10.1038/s41598-023-30201-1 (PMC9950079; doi:10.1038/s41598-023-30201-1)
Supplement: Supplementary file 2 — Supplementary Information 2. [file 41598_2023_30201_MOESM2_ESM.docx]

# Investigating responses to object-labels in the domestic dog (*Canis familiaris*)

**Hanna Kőszegi^a,b^, Claudia Fugazza^a,h^, Lilla Magyari^a,c,d^, Ivaylo Borislavov Iotchev*^a,e^, Ádám Miklósi^a,h^, Attila Andics^a,f,g,h^**

a ELTE, Eötvös Loránd University, Department of Ethology, Pázmány Péter sétány 1/c, Budapest, 1117, Hungary

b University of Veterinary Medicine, Department of Animal Breeding, Nutrition and Laboratory Animal Science, István utca 2, Budapest 1078, Hungary

c Department of Social Studies, Faculty of Social Sciences, University of Stavanger, Norway

d Centre for Reading Education and Research, Faculty of Arts and Education, University of Stavanger, Stavanger, Norway

e MTA-ELTE “Lendület” Companion Animal Research Group, Hungarian Academy of Sciences – Eötvös Loránd University, 1117 Budapest, Pázmány Péter sétány 1/c, Hungary

f MTA-ELTE “Lendület” Neuroethology of Communication Research Group, Hungarian Academy of Sciences – Eötvös Loránd University, 1117 Budapest, Pázmány Péter sétány 1/c, Hungary

g ELTE NAP Canine Brain Research Group, 1117 Budapest, Pázmány Péter sétány 1/c, Hungary

h ELTE NAP Comparative Ethology Research Group, 1117 Budapest, Pázmány Péter sétány 1/c Hungary

*corresponding author: Ivaylo Borislavov Iotchev, ivaylo.iotchev@gmail.com

**Supplementary Table S1**

| **Name** | **Age**  **(year)** | **Breed** | **Object-name “A”** | **Object-name “B”** |
| --- | --- | --- | --- | --- |
| Zadar | 11 | *Weimaraner* | Papucs (Slipper) | Labda (Ball) |
| Barack | 2 | *Poodle* | Zokni (Sock) | Papucs (Slipper) |
| Bilbo | 4 | *Poodle* | Sün (Hedgehog) | Labda (Ball) |
| Walter | 7 | *Golden retriever* | Papucs (Slipper) | Törcsi (Towel) |
| Borisz | 3 | *Toy pinscher* | Labda (Ball) | Rákocska (Crab) |
| Zsombék | 5 | *Mudi* | Frizbi (Frisbee) | Labda (Ball) |
| Oslo | 2 | *Australian shepherd* | Giraffe (Giraffe) | Balle (Ball) |
| Ezra | 2 | *Border collie mix* | Kötél (Rope) | Zacskó (Bag) |
| Léna | 7 | *Hungarian vizsla – smooth haired* | Póráz (Leash) | Papucs (Slipper) |
| Roy | 6 | *Australian shepherd* | Labda (Ball) | Szarvas (Deer) |
| Dönci | 6 | *Mudi* | Karika (Hoop) | Hangos foci (Loud football?) |
| Borka | 3.5 | *Hungarian vizsla – smooth haired* | Dínó (Dinosaur) | Picsirke (Chicken?) |
| Max | 8 | *Mix* | Cica (Kitty) | Kacsa (Duck) |
| Túró | 3.5 | *English cocker spaniel* | Patkány (Rat) | Labda (Ball) |
| Kajla | 4 | *Australian shepherd* | Frizbi (Frisbee) | Labda (Ball) |
| Borzas | 8 | *Labradoodle* | Cipő (Shoe) | Póráz (Leash) |
| Demi | 6 | *White swiss shepherd dog* | Zokni (Sock) | Papucs (Slipper) |
| Joya | 8 | *Labradoodle* | Kosár (Basket) | Cipő (Shoe) |
| Nara | 3 | *German shepherd* | Csont (Bone) | Labda (Ball) |
| Bingó | 3 | *Mix* | Csont (Bone) | Labda (Ball) |

**Subject table**

**Supplementary Table S2. Condition order.**

|  | **Name** | **Ses1** | **Ses2** | **Ses3** | **Ses4** |
| --- | --- | --- | --- | --- | --- |
| **1** | Zadar | Panel  Fetch | No panel  Look | No panel Fetch | Panel Look |
| **2** | Barack | No panel  Fetch | Panel  Look | Panel Fetch | No panel Look |
| **3** | Bilbo | Panel  Look | No panel  Fetch | No panel  Look | Panel  Fetch |
| **7** | Walter | Panel  Look | No panel  Fetch | No panel  Look | Panel  Fetch |
| **8** | Borisz | No panel  Look | Panel  Fetch | Panel  Look | No panel  Fetch |
| **9** | Zsombék | Panel  Fetch | No panel  Look | No panel  Fetch | Panel  Look |
| **13** | Oslo | No panel  Fetch | Panel  Look | Panel  Fetch | No panel  Look |
| **14** | Ezra | Panel  Look | No panel  Fetch | No panel  Look | Panel  Fetch |
| **15** | Léna | Panel  Fetch | No panel  Look | No panel  Fetch | Panel  Look |
| **16** | Roy | No panel  Fetch | Panel  Look | Panel  Fetch | No panel  Look |
| **17** | Dönci | Panel  Fetch | No panel  Look | No panel Fetch | Panel  Look |
| **18** | Borka | No panel  Fetch | Panel  Look | Panel  Fetch | No panel  Look |
| **19** | Max | Panel  Look | No panel  Fetch | Panel Fetch | No panel  Look |
| **20** | Túró | Panel  Look | No panel  Fetch | No panel  Look | Panel  Fetch |
| **21** | Kajla | No panel  Fetch | Panel  Look | Panel  Fetch | No panel  Look |
| **22** | Borzas | No panel  Fetch | Panel  Look | Panel  Fetch | No panel  Look |
| **23** | Demi | Panel  Fetch | No panel  Look | No panel  Fetch | Panel  Look |
| **24** | Joya | No panel  Fetch | Panel  Look | Panel  Fetch | No panel  Look |
| **25** | Bingó | No panel  Look | Panel Fetch | Panel  Look | No panel  Fetch |
| **26** | Nara | No panel  Look | Panel  Fetch | Panel  Look | No panel  Fetch |

Video S1. Demonstration of the experimental set-up and behaving dog in the fetching response condition.

Video S2. Demonstration of the experimental set-up and behaving dog in the looking response condition.
